# Accuracy and User-Acceptability of HIV Self-Testing Using an Oral Fluid-Based HIV Rapid Test

**DOI:** 10.1371/journal.pone.0045168

**Published:** 2012-09-17

**Authors:** Oon Tek Ng, Angela L. Chow, Vernon J. Lee, Mark I. C. Chen, Mar Kyaw Win, Hiok Hee Tan, Arlene Chua, Yee Sin Leo

**Affiliations:** 1 Department of Infectious Disease, Tan Tock Seng Hospital, Singapore; 2 Department of Clinical Epidemiology, Tan Tock Seng Hospital, Singapore; 3 Department of Epidemiology and Public Health, National University of Singapore, Singapore; 4 Department of Sexually Transmitted Infection Control, National Skin Centre, Singapore; McGill University Health Centre, McGill University, Canada

## Abstract

**Background:**

The United States FDA approved an over-the-counter HIV self-test, to facilitate increased HIV testing and earlier linkage to care. We assessed the accuracy of self-testing by untrained participants compared to healthcare worker (HCW) testing, participants’ ability to interpret sample results and user-acceptability of self-tests in Singapore.

**Methodology/Principal Findings:**

A cross-sectional study, involving 200 known HIV-positive patients and 794 unknown HIV status at-risk participants was conducted. Participants (all without prior self-test experience) performed self-testing guided solely by visual instructions, followed by HCW testing, both using the OraQuick ADVANCE Rapid HIV 1/2 Antibody Test, with both results interpreted by the HCW. To assess ability to interpret results, participants were provided 3 sample results (positive, negative, and invalid) to interpret. Of 192 participants who tested positive on HCW testing, self-testing was positive in 186 (96.9%), negative in 5 (2.6%), and invalid in 1 (0.5%). Of 794 participants who tested negative on HCW testing, self-testing was negative in 791 (99.6%), positive in 1 (0.1%), and invalid in 2 (0.3%). Excluding invalid tests, self-testing had sensitivity of 97.4% (95% CI 95.1% to 99.7%) and specificity of 99.9% (95% CI: 99.6% to 100%). When interpreting results, 96%, 93.1% and 95.2% correctly read the positive, negative and invalid respectively. There were no significant demographic predictors for false negative self-testing or wrongly interpreting positive or invalid sample results as negative. Eighty-seven percent would purchase the kit over-the-counter; 89% preferred to take HIV tests in private. 72.5% and 74.9% felt the need for pre- and post-test counseling respectively. Only 28% would pay at least USD15 for the test.

**Conclusions/Significance:**

Self-testing was associated with high specificity, and a small but significant number of false negatives. Incorrectly identifying model results as invalid was a major reason for incorrect result interpretation. Survey responses were supportive of making self-testing available.

## Introduction

Early awareness of HIV status is crucial to prevent onward transmission and achieve favorable treatment outcomes [1], [2]. A recent randomized trial demonstrating a 96% reduction in onward transmission associated with antiretroviral therapy further highlights the importance of early diagnosis, a prerequisite for treatment initiation [3]. However, late diagnosis of HIV remains a major public health issue, contributed by the fact that many at- risk persons do not seek testing at HIV test sites [4]–[6].

Self-testing using oral fluid-based rapid tests has received support from activists and public health officials as a possible means of increasing testing rates and awareness of HIV serostatus [7], [8]. Prior experience with home testing has demonstrated that certain at-risk individuals prefer testing in private. In the first year of home testing availability, 174,316 home-tests were ordered in the United States [9]. This was despite the inconvenience and discomfort of finger-prick to obtain dried-blood spots and having to mail these samples to a commercial company before receiving results via phone-call after a few days. 0.9% of the tests were HIV-positive, 3 times the estimated national prevalence. Oral fluid self-testing would minimize discomfort and enable users to obtain results immediately.

In Singapore, late diagnosis of HIV remains a major public health issue. In 2011, 461 new cases of HIV were reported to the Singapore Ministry of Health (Singapore MOH), bringing the number of people living with HIV to 3,813 [10]. The predominant mode of transmission was sexual, with 46% reporting heterosexual transmission risk, 42% homosexual risk, and 9% bisexual risk. Similar to previous years, 53% had AIDS on initial diagnosis. Of the new cases in 2011, 58% were diagnosed while in medical care, with only 28% diagnosed during health screening or voluntary screening.

In the last 5 years, the Singapore MOH has aggressively expanded HIV testing by increasing the number of anonymous test sites, making HIV oral rapid tests available at HIV test sites, and offering routine opt-out HIV testing for all inpatients admitted into public hospitals [11]. The recommendations for routine opt-out inpatient screening followed the release of revised United States Centers for Disease Control guidelines on HIV testing in 2006 [12]. Despite these measures, the prevalence of HIV late-presentation in Singapore remains unchanged.

While the United States Food and Drug Administration (US FDA) has recently approved the OraQuick In-Home HIV Test for over-the-counter sale, HIV self-tests remain illegal in Singapore [13]. A previous study examining blood-based self-testing among 420 individuals in Singapore revealed poor test performance by untrained persons, and difficulty in test interpretation [14]. Sixty-seven percent of participants reported blood sampling and transfer as the most difficult step. Preliminary results using an oral-fluid HIV test in Singapore demonstrated improved test accuracy and interpretation, compared to the blood-based kit [15].

Understanding the accuracy and acceptability of HIV self-testing in Singapore would inform deliberations on HIV self-testing by the Singapore Health Sciences Authority, the regulatory body for medical diagnostics in Singapore. We studied 994 untrained participants to determine the accuracy and feasibility of HIV self-testing compared to tests performed by trained healthcare workers. This study aims were to determine (1) the sensitivity and specificity of self-testing by untrained persons compared with healthcare worker testing, (2) the ability of untrained persons to accurately interpret sample test results and (3) user attitudes towards oral fluid-based self-testing.

## Methods

### Ethics Review

This study was approved by the National Healthcare Group ethics review board. Written informed consent was obtained from all study participants. All study healthcare workers received Singapore Ministry of Health accredited training on point-of-care HIV rapid testing.

### Setting and Design

From December 2008 to August 2010, a cross-sectional study was conducted at 4 HIV test sites in Singapore: the Communicable Disease Centre (CDC) outpatient clinic (Singapore’s reference HIV treatment centre), the Department of Sexually Transmitted Infections (STI) Control (DSC) clinic (Singapore’s reference STI treatment centre) and two private general practice clinics. These represent a diverse spectrum of potential HIV self-test end-users in Singapore.

### Study Population

The DSC, which provides point-of-care rapid testing to STI patients, and the two general practice clinics, which were government-approved anonymous point-of-care HIV rapid test sites, were included to recruit “at-risk” participants who did not yet know their HIV status. As new HIV diagnoses were expected to be rare among at-risk participants, we also recruited known HIV-positive participants from the CDC, the national HIV treatment centre, to facilitate evaluation of self-testing among confirmed HIV-seropositive individuals. Additionally, any effect of sociodemographic differences between known HIV-seropositive individuals and at-risk individuals on study endpoints would be detected.

As there were two trained healthcare workers recruiting from 4 study sites, convenience sampling was conducted at the sites when the healthcare workers were available. There was no predefined order for the 2 healthcare workers to rotate to the various sites. Additionally, at CDC and DSC, sampling of one participant each from blocks of 2 was performed, with pre-determined random selection of the order to be selected in each pair. Due to smaller numbers of at-risk attendees, all at-risk persons seeking rapid HIV tests at the private clinics whenever the healthcare workers were available were approached to participate. Only consenting individuals aged 21 years and older who had never undergone HIV rapid testing were recruited.

### Study Procedures

Pre-test counseling, as per Singapore Ministry of Health training on point-of-care HIV rapid testing, was provided immediately following study consent. Following pre-test counseling, participants were provided a 31-question pre-test survey on demographics and knowledge and attitudes toward HIV self-testing (Appendix S1). For English-illiterate participants (defined as self-reporting unable to read or write English), pre- and post-test surveys were administered verbally by the trained healthcare worker, using either Mandarin or Malay, to the participant (the other two main languages in Singapore). English-illiterate participants were included to determine if English literacy would be a factor in test accuracy or interpretation. To determine sensitivity and specificity comparing self-testing by untrained persons to healthcare worker testing (reference standard), participants performed self-testing guided solely by an 11-step pictorial guide, followed by a repeat test by a trained healthcare worker.

The OraQuick ADVANCE Rapid HIV 1/2 Antibody Test (OraSure Technologies, Bethlehem, Pennsylvania, USA), an approved point-of-care rapid test in Singapore, was used. Our study kit included all commercial kit testing equipment and an 11-step pictorial instruction sheet with English instructions designed by the study team based on test kit instructions, which replaced the product insert (Appendix S2). These 11 steps included test kit preparation (Steps #1–6), collection of oral fluids (#7–8), insertion of the specimen into the reagent vial (#9, 10), and interpretation of results (#11). Test results were read based on the presence or absence and position of 2 reddish-purple lines in the result window. An instruction sheet with 7 pictures representing the range of results was provided for test result interpretation. The pre- and post-test questionnaires and information sheets were modeled on those used in a prior study [14].

To reduce bias from verbal instructions by the healthcare worker, no verbal instructions were given in the course of self-testing. Both test results (the first by self-testing, and the second by healthcare worker testing) were interpreted by the healthcare worker. To determine the ability of untrained persons to accurately interpret test results, participants were then provided 3 model test results (positive, negative and an invalid result) to interpret. Interpretation of model test results were based on the reference pictorial guide provided. Finally, a 14-question post-test survey was administered (Appendix S1). Results of self-test and healthcare worker conducted test, both read by the healthcare worker, were revealed only after the participant had interpreted the model tests and completed the post-test survey. All at-risk participants received pre and post-test counseling and standard-of-care confirmatory HIV blood test, according the MOH guidelines [16]. Post-test counseling, as per Singapore Ministry of Health training on point-of-care HIV rapid testing, was conducted following completion of all study procedures. CDC participants did not undergo additional HIV blood tests and were counseled that the self-testing results would not alter their HIV status.

### Statistical Analysis

Key demographic variables were compared between at-risk individuals and known HIV-positive patients, using the chi-square test to compare proportions for categorical variables, and the Mann-Whitney test to compare medians for continuous variables. Inter-rater agreement, measured by the κ value, was estimated in two analyses – one including invalid results, and one excluding invalid results. For the analysis of self-testing sensitivity and specificity, pairs with invalid test results were excluded. To assess the variability in accuracy estimates due to invalids, in a second analysis, the 3 invalid results were then assumed to be discordant pairs. This was as sensitivity and specificity estimates could only be calculated on dichotomous outcomes for both the test under evaluation (self-test) and the reference test (healthcare worker test). In the analysis of interpretation results, sensitivity analysis was performed, by eliminating incorrect results that were reported as invalid. This was to estimate the proportion of misinterpretations which resulted in a positive or negative result.

Due to presence of zero cells, Fisher’s exact test was used to explore demographic and behavioral factors associated with false negative self-testing. Univariate and multivariate logistic regression analysis was used to explore demographic and behavioural associations with wrongly interpreting a positive or invalid sample result as negative. Fisher’s exact test was used to compare rates of incorrect model result interpretation between known HIV-seropositive individuals and at-risk individuals. Covariates analyzed were gender, age, marital status, ethnicity, highest education attained, monthly income, English literacy and sexual orientation.

Complete case analysis was used for missing data. Analyses were performed on Stata Release 11.1 (Stata Corporation, College Station, Texas, USA).

## Results

There were a total of 994 study participants, of which 200 (20.1%) were known HIV-positive patients at the CDC, 598 (60.2%) were at-risk clients at the DSC clinic and 196 (19.7%) were at-risk clients at the private clinics (Table 1). Participants from all study sites were predominantly male (86.2% to 90%). The median age was 32.4 years (IQR: 27.1 to 40.5). Overall, 67.2% of participants had more than 10 years of formal schooling, 59.5% earned more than USD1,500 and 89.9% were English literate. Heterosexual risk was reported by 83.7%, homosexual risk by 9.5% and bisexual risk by 6.8% of study participants. Compared to at-risk participants, known HIV-positive patients were older, less educated, lower salaried and had less English literacy (all P<0.001). Two (0.3%) at-risk participants were newly diagnosed with HIV infection by ELISA and Western Blot testing – both had positive results on self-testing and healthcare worker testing; bringing the total number of HIV-positive participants to 202 (20.3%).

**Table 1:** Demographic characteristics and transmission risk factor, by site.^a^

Characteristics	CDC (HIV-positive patients) (n = 200),n (column %)	DSC (At-risk participants) (n = 598),n (column %)	Private clinics (At-risk participants) (n = 196),n (column %)	Total (n = 994),n (column %)
Gender (male)	180 (90.0)	531 (88.8)	169 (86.2)	880 (88.5)
Age (median, IQR)	45.3 (36.5 to 51.9)	29.6 (25.5 to 35.1)	35.5 (30.3 to 40.9)	32.4 (27.1 to 40.5)
Current marital status				
Never married	114 (57.3)	385 (64.6)	85 (43.6)	584 (59.0)
Married	67 (33.7)	187 (31.4)	92 (47.2)	346 (34.9)
Divorced/separated/widowed	18 (9.0)	24 (4.0)	18 (9.2)	60 (6.1)
Ethnicity				
Chinese	164 (82.0)	428 (71.6)	173 (88.3)	765 (77.0)
Malay	22 (11.0)	58 (9.7)	3 (1.5)	83 (8.4)
Indian	6 (3.0)	44 (7.4)	10 (5.1)	60 (6.0)
Others	8 (4.0)	68 (11.4)	10 (5.1)	86 (8.7)
Highest education attained				
Did not complete high school	136 (68.0)	169 (28.3)	21 (10.7)	326 (32.8)
Completed high school education	40 (20.0)	210 (35.1)	46 (23.5)	296 (29.8)
College degree and above	24 (12.0)	219 (36.6)	129 (65.8)	372 (37.4)
Monthly income				
<USD 1500	132 (66.0)	230 (38.7)	38 (19.6)	400 (40.5)
USD 1500 to 3000	51 (25.5)	216 (36.4)	54 (27.8)	321 (32.5)
>USD 3000	17 (8.5)	148 (24.9)	102 (52.6)	267 (27.0)
English literate	145 (72.5)	557 (93.1)	192 (98.0)	894 (89.9)
Transmission risk factor				
Heterosexual	126 (63.6)	544 (91.6)	154 (80.2)	824 (83.7)
Homosexual	45 (22.7)	25 (4.2)	23 (12.0)	93 (9.5)
Bisexual	27 (13.6)	25 (4.2)	15 (7.8)	67 (6.8)

a Missing data, which accounted for less than 2% of participants for any question, was omitted from analysis. P-value for all comparisons except gender<0.001. P-value for gender 0.474.

Eight result pairs with missing data for both the self-test and healthcare worker conducted test were excluded (4 known HIV-infected participants and 4 at-risk participants), leaving 986 result pairs for analysis. Of 192 participants who tested positive on HCW testing, self-testing was positive in 186 (96.9%), negative in 5 (2.6%), and invalid in 1 (0.5%). Of 794 participants who tested negative on HCW testing, self-testing was negative in 791 (99.6%), positive in 1 (0.1%), and invalid in 2 (0.3%). The κ value for inter-rater agreement comparing self-testing to healthcare worker testing was 0.97 (95% CI: 0.95–0.99, P<0.001), which showed excellent agreement. In the analysis excluding the 3 invalid self-tests, the corresponding κ value was 0.98 (95% CI: 0.96 to 1.00).

### HIV Self-test Sensitivity and Specificity

For analysis of sensitivity and specificity, the eight result pairs with missing data and 3 invalid self-tests were excluded, leaving 983 result pairs for analysis. Self-testing had a sensitivity of 97.4% (95% CI: 95.1% to 99.7%) and specificity of 99.9% (95% CI: 99.6% to 100%) compared to healthcare worker testing (Table 2). The most common pattern of discordant results was a false-negative self-test paired with a positive healthcare worker test in 5 known HIV-positive participants. Based on healthcare worker assessment of the 5 participants with false-negative results, 4 (80%) had incorrect collection of oral fluid and 2 (40%) had incorrect test kit preparation. In qualitative discussions with study healthcare workers, the commonest error for oral fluid collection was using the collection pad to swab the external lips, instead of the gingival crevice. For incorrect test kit preparation, the commonest error was touching the collection pad during removal from packaging, or spilling the test solutions. There were no independent demographic or behavioral predictors of a false-negative self-test. Due to small numbers, multivariate analysis of predictors of discordant results was not performed.

**Table 2:** Pattern of self-test results, by healthcare worker testing results.^a^

Healthcare worker testing result	Self-testing result	Number (% of total) (total n = 983)
Positive	Positive	186 (96.9%)
	Negative	5 (2.6%)
	Invalid	1 (0.5%)
Total		192 (100%)
Negative	Negative	791 (99.6%)
	Positive	1 (0.1%)
	Invalid	2 (0.3%)
Total		794 (100%)

a Three invalid self-tests were excluded from analysis, of which 1 was paired with a positive healthcare worker test and another 2 paired with a negative healthcare worker test. Eight result pairs with missing data were also excluded from analysis.

Sensitivity and specificity were re-estimated with the 3 test-pairs with invalid results assumed to be discordant. The estimates were minimally affected. Self-testing had a sensitivity of 96.9% (95% CI: 94.4% to 99.4%) and specificity of 99.6% (95% CI: 99.1% to 100%).

### Model Test Result Interpretation

Positive, negative and invalid sample tests were interpreted correctly by 949 (96.0%), 925 (93.1%) and 939 (95.2%) participants respectively (Table 3). The commonest pattern of misinterpretation was interpreting a negative sample result as invalid, a finding in 61 (6.1%) of participants. Five (0.5%) of participants misinterpreted a positive sample result as negative and 29 (2.9%) misinterpreted an invalid result as negative. Thirty-two participants (3.2%) wrongly interpreted a positive or invalid sample result as negative. Compared to at-risk participants, a higher proportion of known-HIV positive participants misinterpreted model positive (91.0% vs. 97.2%, P<0.001), negative (85.5% vs. 95.0%, P<0.001), and invalid results (91.9% vs. 96.1%, P = 0.023). There were no independent demographic or behavioral predictors of wrongly interpreting a positive or invalid sample result as negative.

**Table 3:** Participant interpretation of sample test results, by site.^a^

	CDC (n = 200),n (column %)	DSC (n = 598),n (column %)	Private clinics (n = 196),n (column %)	Total (n = 994),n (column %)
Positive sample result^b^				
Read correctly	182 (91.0)	579 (96.8)	188 (98.4)	949 (96.0)
Read as negative	4 (2.0)	1 (0.2)	0 (0)	5 (0.5)
Read as invalid	14 (7.0)	18 (3.0)	3 (1.6)	35 (3.5)
Negative sample result^b^				
Read correctly	171 (85.5)	568 (95.0)	186 (94.9)	925 (93.1)
Read as positive	1 (0.5)	5 (0.8)	2 (1.0)	8 (0.8)
Read as invalid	28 (14.0)	25 (4.2)	8 (4.1)	61 (6.1)
Invalid result^c^				
Read correctly	182 (91.9)	585 (97.8)	172 (90.5)	939 (95.2)
Read as negative	10 (5.1)	5 (0.8)	14 (7.4)	29 (2.9)
Read as positive	6 (3.0)	8 (1.3)	4 (2.1)	18 (1.8)
Analyses excluding invalids				
Positive sample result	CDC (n = 186),n (column %)	DSC (n = 580),n (column %)	Private clinics (n = 188),n (column %)	Total (n = 954),n (column %)
Read correctly	182 (97.8)	579 (99.8%)	188 (100%)	949 (99.5%)
Read as negative	4 (2.2)	1 (0.2%)	0 (0%)	5 (0.5%)
Negative sample result	CDC (n = 172),n (column %)	DSC (n = 573),n (column %)	Private clinics (n = 188),n (column %)	Total (n = 933),n (column %)
Read correctly	171 (99.4)	568 (99.1)	186 (98.9)	925 (99.1)
Read as positive	1 (0.6)	5 (0.9)	2 (1.1)	8 (0.9)

a Five positive samples and 6 invalid were missing interpretation results and excluded from analysis.

b P-value comparing incorrect interpretation between known HIV-positive participants and others was <0.001.

c P-value comparing incorrect interpretation between known HIV-positive participants and others was 0.023.

In the analysis excluding tests reported as invalid, the rates of correct interpretation improved, with 99.5% of participants and 99.1% of participants correctly identifying the model positive and negative sample respectively.

### Survey Results on User Acceptability

The questionnaire revealed that 56.4% of participants had heard of HIV rapid tests, with the highest awareness among private clinic at-risk participants (83.2%) (Table 4). More than 90% of all participants preferred to receive their test results within one hour. Overall, 87.4% of participants would purchase an over-the-counter rapid test kit, with the highest proportion being among private clinic at-risk participants (92.4%). 89% wanted to conduct HIV testing in private and only 36.4% would agree to have their names recorded when undergoing a HIV test. 72.5% felt that pre-test counseling was necessary.

**Table 4:** Selected participants’ opinions regarding self-testing before and after self-testing, by site.^a^

	CDC (HIV-positive participants) (n = 200),n (column %)	DSC (At-risk participants) (n = 598),n (column %)	2 Private Clinics (At-risk participants) (n = 196),n (column %)	Total (n = 994), n (column %)
Pre-Test Opinions				
Heard about HIV rapid test before	118 (59.0)	279 (46.7)	163 (83.2)	560 (56.4)
Like to know HIV test results in less than 1 hour	182 (91.0)	579 (96.8)	193 (98.5)	954 (96.0)
Would purchase kit over-the-counter from retailoutlets	159 (79.5)	528 (88.4)	181 (92.4)	868 (87.4)
Agree to have name recorded during HIV test	82 (41.2)	258 (43.2)	21 (10.8)	361 (36.4)
Prefer to conduct HIV testing in private	161 (80.5)	532 (89.4)	188 (96.4)	881 (89.0)
Pre-test counseling necessary	134 (67.0)	434 (72.9)	149 (76.8)	717 (72.5)
Post-test opinions				
Test kit instructions were easy to understand	174 (87.4)	581 (97.2)	194 (100.0)	949 (95.8)
Test kit is convenient to use	190 (96.5)	584 (98.2)	182 (98.9)	956 (98.0)
This kit should be sold in public outlets	168 (84.4)	551 (92.3)	165 (85.1)	884 (89.3)
Would recommend this kit to others	171 (85.9)	576 (96.6)	192 (99.0)	939 (94.9)
Post-test counseling is necessary	154 (77.4)	429 (72.5)	152 (80.0)	735 (74.9)
Would pay at least USD15 for this kit	44 (22.1)	151 (25.3)	82 (42.5)	277 (28.0)

a Missing data, which accounted for less than 2% of participants for any question, was omitted from analysis.

Post self-testing, approximately 95% felt that test kit instructions were easy to understand, the kit was convenient to use, and that they would recommend it to others. Accordingly, almost 90% felt that the kits should be available over-the-counter. 74.9% felt that post-test counseling was necessary. Only 28% would pay at least USD15 for the kit with the highest proportion being among private clinic at-risk participants (42.5%).

## Discussion

In our study population with differing educational attainments and socio-economic backgrounds, observed self-testing by untrained users had excellent specificity (99.9%, 95% CI: 99.6% to 100%), with lower sensitivity (97.4% (95% CI: 95.1% to 99.7%). The corresponding false-negative rate is 2.6%. Only 3 (0.3%) of 986 self-tests were invalid. In evaluating the participants’ ability to interpret model test results, a minority misinterpreted positive and negative results, and of these, most were misread as invalid. In agreement with most published studies, questionnaire responses revealed a high level of user support for oral fluid-based HIV self-testing.

### Sensitivity and Specificity of HIV Self-testing

Our study suggests that a HIV oral-fluid self test has markedly better accuracy compared to a blood-based self-test in Singapore. In a previous study by our group, blood-based self-testing had a κ of 0.277. Out of 350 self-tests conducted, 197 (56.3%) were invalid in the prior study. In our current study, the κ value for inter-rater agreement comparing self-testing to healthcare worker testing was 0.97 (95% CI: 0.95–0.99, P<0.001). This suggests that, in Singapore, participants are much more able to perform oral fluid sample collection compared to finger-prick based sample collection.

Our sensitivity and specificity results, and number of invalid tests, are similar to previous studies involving observed self-testing using oral-fluid HIV tests. In an observed use regulatory study submitted to the US FDA involving 531 known HIV positives and 500 persons of unknown status, HIV oral-fluid self-test sensitivity was 97.9% (95% CI:95.0% to 99.4%), and specificity 99.79% (95% CI: 98.1% to 100.0%) [17]. A new metric, the Test System Failure Rate (TFR), measured the rate of self-tests with neither a positive or negative result (thereby including invalids). The overall TFR in the above observed study was 3.3%, compared to 0.3% in our study. In a study involving 478 emergency department attendees who conducted self-testing using oral fluid or blood-based tests, Gaydos et al. in Baltimore determined a HIV self-testing accuracy of 99.6% [18]. Ninety-one percent of self-tests were conducted using an oral-fluid kit. In a study of 260 participants, who received a brief demonstrating of oral-fluid testing and then conducted supervised oral HIV self-testing in Blantyre, Malawi, Choko et al. determined a HIV self-test accuracy of 99.2% with two false negative tests [19].

Incorrect oral fluid collection is a potential factor in false negative results. Four of the 5 participants in our study with false-negative results were observed to incorrectly perform oral fluid collection. For accurate results, it is important that oral mucosal transudate be collected from the crevicular space between the gums and teeth as this fluid is rich in immunoglobulin G (IgG), the main class of antibodies detected for a positive HIV test [20]. Sample collection should avoid the cheeks, floor and roof of the mouth, as saliva has low IgG levels and contains bacterial and salivary proteases which may degrade IgG [21]. Another reason for false-negative results could be decreased anti-gp41 antibody titres among known HIV positive participants on treatment. This could affect test results as the oral fluid-based rapid test used in this study detects anti-gp41 antibodies [22]. The current product insert categorically warns known HIV positive patients not to use the test [17].

### Accuracy of Model Result Interpretation

Accuracy of model result interpretation in our study was very similar to a device interpretation study reported to the FDA for self oral-fluid HIV test approval. In the regulatory study, the rates of correctly identifying model results were: high positive model tests 95.00%, negative results 93.80% and invalid results 92.10% [17]. In analysis excluding results interpreted as invalid in the regulatory study, 96.74% and 95.57% of participants accurately identified high positive and negative results respectively. This suggests that emphasizing seeking re-testing at an anonymous HIV test site should a user read a result as invalid would significantly help mitigate errors in interpretation in the course of actual use of a home self-test.

When interpreting test results, false positive interpretation could result in unnecessary anxiety, while false negative interpretation could result in an erroneous sense of security and continued high-risk behavior [7], [23]. The majority of interpretation errors were positive and negative results read as invalid. The implication of this error could be limited by including in test instructions that patients who test positive or invalid should present to HIV test sites for confirmation. The effects of a false negative interpretation are harder to mitigate. A potential solution to reduce misinterpretation while maintaining test confidentiality could be to use existing mobile communication technology to capture and transmit a digital image of the self-test result for digital or visual verification by a computer or trained person respectively. The verified result could be conveyed to the self-tester via text message or phone-call [24].

### Survey Results

In agreement with most published studies, questionnaire responses revealed a high level of user support for oral fluid-based HIV self-testing. The majority of respondents (>85%) would purchase over-the-counter test kits which provide results within an hour, liked anonymous testing in private, and would recommend the kit to others. In surveys from California and Seattle representing diverse populations, up to a third of respondents were supportive of self-testing [25]–[27]. In a previous self-test study in Singapore involving a blood-based kit, 89% of at-risk persons preferred testing in private and 97% would recommend the kit to others [14].

However, the current study supports the concern that poorer at-risk populations may not access HIV self-testing [28]. Private clinic participants, the wealthiest group among the test sites, had the highest level of positive responses in support of self-testing, and were more likely to agree to pay at least USD15 for the test kit (the approximate laboratory testing costs currently). If HIV self-testing is to be employed, measures to decrease the financial burden of self-testing warrant consideration.

Although 89% of participants favored testing in private, about three quarters wanted pre- and post-test counseling. One possible measure to provide counseling and linkage to care while maintaining confidentiality would be the use of phone-based counseling [29]. The results from a study of the first 175,000 users of anonymous home collection HIV tests are reassuring – of 400 newly diagnosed HIV clients, telephone counseling and release of results was associated with 74% accepting referral for care, and the rest refusing because they had an existing healthcare provider [9].

### Implications for Possible HIV Self-testing in Singapore

In Singapore, HIV self-testing can be operationalized as an extension of the currently available MOH-approved anonymous HIV test sites. Anonymous test sites are an exception to the legal requirement to report all HIV positive tests to a name-based MOH registry. Unlike provider-based anonymous testing, alternative forms of pre and post-test counseling and linkage to care would obviously be necessary for self-testing. As discussed above, phone based counseling is a potential option. In the United States, the commercial company marketing the FDA approved HIV self-test has committed to maintaining a 24-hour toll-free number providing counseling, advice on test conduct, and referral services for care. This strategy could be used in Singapore.

### Limitations

A major limitation of our study was that test assessment and interpretation was conducted in an observed setting. This could introduce the potential for overestimation of accuracy as participants could have additional information otherwise not available in private. In the United States, the follow on study of unobserved self-testing submitted to the FDA involving 5558 participants reported a lower sensitivity of 93.0% (95% CI: 86.6% to 96.9%) compared to the observed testing study. A similar study in the Singapore context would further strengthen estimates of accuracy in actual practice. A related limitation was known HIV-positive participants may be less invested in self-test results, and bias sensitivity estimates lower. However, the above unobserved study in patients on unknown status did not further improve sensitivities appreciably. It is likely that HIV-positive participants were less invested in accurately interpreting model test results, as misinterpretation was significantly more prevalent among HIV-positive participants compared with the at-risk population. Selection bias could have occurred as convenience sampling was employed. The questionnaire responses of known HIV-positive participants may be influenced by healthcare provision and education but this was unlikely to affect the ability to perform and interpret tests as all participants had no prior experience with HIV rapid tests. Issues relevant to self-testing not addressed by the current study include the psychological impact of lack of face-to-face counseling, the effect on surveillance data collection, and measures to prevent abuse of over-the-counter availability of self-tests (for example, the use of such tests to coerce individuals to undergo HIV testing).

Self-testing was associated with high specificity, and a small but significant number of false negatives. Incorrectly identifying model results as invalid was a major reason for incorrect result interpretation. Survey responses demonstrated strong support for making self-testing available. In Singapore, HIV self-testing could be operationalized as an extension of the currently available anonymous HIV test sites. Further studies in unobserved use could aid in the discussion on making self-testing available in Singapore.

## Supporting Information

Appendix S1 **Oral Rapid Test Questionnaire.**
(PDF)

Appendix S2 **Patient Instruction Sheet.**
(PDF)
