# Supplementary material for: Accuracy and User-Acceptability of HIV Self-Testing Using an Oral Fluid-Based HIV Rapid Test
Source: PLoS One. 2012 Sep 17;7(9):e45168. doi: 10.1371/journal.pone.0045168 (PMC3444491; doi:10.1371/journal.pone.0045168)
Supplement: Appendix S1 — Oral Rapid Test Questionnaire. (PDF) [file pone.0045168.s001.pdf]

Tan Tock Seng HOSPITAL

HIV Rapid Test Questionnaire (PRE-TEST)

Subject Study ID [ ]

Please tick ☑ only one choice unless specified

Q1 Gender
☐ Male (1) ☐ Female (2)

Q2 What is your date of birth? [ ][ ] / [ ][ ][ ][ ] (mm/yyyy)

Q3 What is your current marital status?
☐ Never married (1) ☐ Married (2)
☐ Divorced (3) ☐ Separated (4)
☐ Widowed (your spouse has passed away) (5)

Q4 What is your nationality?
☐ Singapore citizen (1) ☐ Singapore PR (2)
☐ Foreigner (3), please specify **Country of origin** [ ] and **Length of stay in SG (in years)** [ ] . [ ]

Q5 What is your religion?
☐ No religion (0) ☐ Buddhism (1)
☐ Christianity (2) ☐ Catholic (3)
☐ Islam (4) ☐ Taoism (5)
☐ Others (6) please specify [ ]

Q6 What is your ethnic group?
☐ Chinese (1) ☐ Malay (2)
☐ Indian (3) ☐ Others (4) please specify [ ]

Q7 What is your highest educational level?
☐ Primary (PSLE or less) (1) ☐ Secondary ('O' or 'N' Level) (2)
☐ ITE certificate (3) ☐ Diploma / 'A' Level (4)
☐ Degree and above (5)

Q8 What is your last occupation? [ ]

Q9 What was your last drawn monthly income (in Singapore dollars)?
☐ Less than $1000 (0) ☐ $1000 - $1999 (1)
☐ $2000 - $2999 (2) ☐ $3000 - $3999 (3)
☐ $4000 - $4999 (4) ☐ $5000 - $5999 (5)
☐ $6000 and above (6)

Q10 Are you currently employed?
☐ No (0) ☐ Yes (1)

Q11 Which languages can you <u>speak?</u> (tick all that apply)
☐ English (1) ☐ Mandarin (2)
☐ Malay (3) ☐ Tamil (4)
☐ Local dialects (Teochew, Hokkien, Hakka, Cantonese, etc) (5)
☐ Others (6), please specify [ ]

Q12 Which languages can you <u>read?</u> (tick all that apply)
☐ None (0) ☐ English (1)
☐ Chinese (2) ☐ Malay (3)
☐ Tamil (4)
☐ Others (5), please specify [ ]

Q13 Do you think HIV/AIDS is a serious disease?
☐ Very serious (1) ☐ Serious (2)
☐ Somewhat serious (3) ☐ Not serious at all (4)

Tan Tock Seng HOSPITAL

HIV Rapid Test Questionnaire (PRE-TEST)

Subject Study ID [ ]

Please tick ☑ only one choice unless specified

Q14 **Have you had a HIV/AIDS test done before?**
- ☐ No (0)
- ☐ Yes (1)
- ☐ Not sure / cannot remember (99)

If "Yes" answer Q14a to Q14c, if "No" answer Q14d, if "Not sure" skip to Q15.

If YES

Q14a **When** [|] / [|] (mm/yy) **and where** [ ] **(the location) did you last do the test**

Q14b **What were your reason(s) for doing the HIV test? (tick all that apply)**
- ☐ I wanted to, because it was possible that I had been infected with HIV/AIDS (1)
- ☐ My partner asked me to test (2)
- ☐ I'm required to have the test (employment, army etc) (3)
- ☐ Others (4), please specify [ ]

Q14c **Did you find out the result of your test?**
- ☐ No (0), if no why not? [ ]
- ☐ Yes (1)

If NO

Q14d **If you did not have a HIV/AIDS test before, why did you not get tested? (tick all that apply)**
- ☐ I thought that I had a low chance of getting HIV/AIDS (1)
- ☐ Too expensive (2)
- ☐ Takes too much time / inconvenient (3)
- ☐ I am afraid of knowing that I may have HIV/AIDS (4)
- ☐ There is no cure so it doesn't matter (5)
- ☐ I don't know where to get tested (6)
- ☐ I'm afraid that my results may be made known to other people (7)
- ☐ Others (8) please specify [ ]

Q15 **What do you think are the benefits of HIV/AIDS testing? (tick all that apply)**
- ☐ I won't have to worry, if my results were negative (1)
- ☐ Get early treatment, if HIV positive (2)
- ☐ Protecting my regular partners (Wife, husband, or long-term partner) (3)
- ☐ Protecting my other sexual partners (not regular partner) (4)
- ☐ Others (5) specify [ ]

Q16 **What do you think is your chance of getting HIV/AIDS?**
- ☐ High chance (1)
- ☐ Some chance (2)
- ☐ Very low chance (3)
- ☐ No chance (4)

Q17 **Who have you had sex with?**
- ☐ Women only (1)
- ☐ Men only (2)
- ☐ Usually women (3)
- ☐ Usually men (4)

Q18 **Who were your partners in the past year? (tick all that apply)**
- ☐ Regular partners (Wife, husband, or long-term partner) (1)
  How many? [ ]
- ☐ Commercial sex workers (Sex in exchange for money) (2)
  How many? [ ]
- ☐ Non-regular (Not regular, and did not pay for sex e.g. one night stand) (3)
  How many? [ ]

HIV Rapid Test Questionnaire (PRE-TEST)

Subject Study ID

Please tick ☑ only one choice unless specified

Q19	**How often do you use a condom when having sex (vaginal, oral, and anal included) in the last 6 months?**	☐ Always (1)<br>☐ Sometimes (less than half the time) (3)	☐ Usually (more than half the time) (2)<br>☐ Never (4)
Q20	**Have you ever had a STD (sexually transmitted disease) such as syphilis, herpes, gonorrhoea, etc?**	☐ No (0)<br>☐ Don't know, not sure (99)	☐ Yes (1)
Q21	**Have you heard of a HIV rapid test (test that gives results in less than one hour) before this session?**	☐ No (0)	☐ Yes (1)
Q22	**Would you want to know your HIV test results in less than 1 hour if possible?**	☐ No (0)	☐ Yes (1)
Q23	**If you can buy this rapid test from the supermarket or shops, would you get it and do the test on your own?**	☐ No (0)	☐ Yes (1)

If YES to Q23

Q23a **Why? (tick all that apply)**

- ☐ I won't have to worry, if my results were negative (1)
- ☐ Get early treatment, if HIV positive (2)
- ☐ Protect my regular partners (Wife, husband, or long-term partner) (3)
- ☐ Protect my other sexual partners (not regular partner) (4)
- ☐ It can be done privately and no one else will know the results (5)
- ☐ Such tests are as accurate as a confirmatory test (6)
- ☐ Others (7), please specify ________

If NO to Q23

Q23b **Why not? (tick all that apply)**

- ☐ I already know my results (1)
- ☐ I am afraid to know my results (2)
- ☐ There is no cure for HIV/AIDS, so it makes no difference (3)
- ☐ I am not sure if the test is accurate (4)
- ☐ Others (5), please specify ________

Q24	**As you know, the rapid test is only a preliminary test (with 99% accuracy). To be 100% sure of your result, a confirmatory test (which gives results in 2 weeks) is needed. If you have done a rapid test on your own, would you still go to a clinic for a confirmatory test?**	☐ No (0)	☐ Yes (1)
Q25	**Do you think confidential counselling by trained healthcare worker is necessary before a HIV/AIDS test?**	☐ No (0)	☐ Yes (1)

If Yes to Q25

Q25a **Why? (tick all that apply)**

- ☐ Allows me to decide if I still want to be tested (1)
- ☐ Explain the seriousness of doing the test and knowing the results (2)
- ☐ Explain why the test is necessary (3)
- ☐ Healthcare workers are knowledgeable (4)
- ☐ Others (5), please specify ________

Tan Tock Seng HOSPITAL

HIV Rapid Test Questionnaire (PRE-TEST)

Subject Study ID

Please tick ☑ only one choice unless specified

If NO to Q25

Q25b	**Why not? (tick all that apply)**	☐ I can handle the results by myself (1)<br>☐ Takes too much time (2)<br>☐ Post-test counselling is enough (3)<br>☐ My personal information may be leaked to others (4)<br>☐ Others (5), please specify	
Q26	**What is the best method for this counselling?**	☐ Direct face to face counselling (1)<br>☐ Over the phone anonymous counselling (2)<br>☐ Written instructions (3)<br>☐ Others (4), please specify	
Q27	**Are you okay with selected healthcare workers seeing your HIV/AIDS results?**	☐ No (0)	☐ Yes (1)
Q28	**Are you okay to have your name and other particulars recorded during a HIV/AIDS test?**	☐ No (0)	☐ Yes (1)
Q29	**Do you prefer to take the HIV/AIDS test in private?**	☐ No (0)	☐ Yes (1)
Q30	**Do you prefer to have a blood or oral (saliva) test for HIV/AIDS, if both are equally accurate?**	☐ Blood (1)<br>☐ No preference (3)	☐ Oral (saliva) (2)
Q31	**How do you feel now, <u>before</u> taking the HIV/AIDS test?**	☐ Very anxious (1)<br>☐ Not anxious at all (3)	☐ A little anxious (2)

Tan Tock Seng HOSPITAL

HIV Rapid Test Questionnaire (POST-TEST)

Subject Study ID

Please tick ☑ only one choice unless specified

Q32	How do you feel now, after taking the HIV/AIDS test?	☐ Very anxious (1) ☐ A little anxious (2) ☐ Not anxious at all (3)
Q33	Did you find the instructions in the test kit easy to understand?	☐ Very easy (1) ☐ Easy (2) ☐ A little difficult (3) ☐ Very difficult (4)
Q34	Did you find the test kit convenient to use?	☐ Very convenient (1) ☐ Convenient (2) ☐ A little inconvenient (3) ☐ Very inconvenient (4)
Q35	Which steps of the test did you find most difficult?	☐ Step 1 – Preparing the kit (1) ☐ Step 2 – Taking the sample (2) ☐ Step 3 – Doing the test (3) ☐ Step 4 – Reading the results (4)
Q36	Are you confident that you have done the HIV rapid test correctly?	☐ No (0) ☐ Yes (1)
Q37	Do you trust the result of the HIV rapid test performed by yourself?	☐ No (0) ☐ Yes (1)
Q38	Do you trust the result of the HIV rapid test performed by the healthcare worker?	☐ No (0) ☐ Yes (1)
Q39	Do you think the HIV rapid test is as accurate as the confirmatory blood test?	☐ No (0) ☐ Yes (1)
Q40	Will knowing your result in less than 1 hour be helpful to you?	☐ No (0) ☐ Yes (1)
Q41	Do you think it is necessary to have a trained counsellor present to support or counsel you of your preliminary results?	☐ No (0) ☐ Yes (1)
	**If YES to Q41**	
Q41a	Why? (tick all that apply)	☐ Explain what the result of the test means (1)<br>☐ Explain why I need a confirmatory test (2)<br>☐ Answer my questions (3)<br>☐ If positive, I need someone to provide support (4)<br>☐ Healthcare workers are knowledgeable (5)<br>☐ Others (6), please specify
	**If NO to Q41**	
Q41a	Why not? (tick all that apply)	☐ I can handle the results by myself (1)<br>☐ Takes too much time (2)<br>☐ Pre-test counselling is enough (3)<br>☐ My personal information may be leaked to others (4)<br>☐ Others (5), please specify
Q42	This test kit needs instructions in the four official languages (English, Malay, Mandarin, and Tamil)?	☐ No (0) ☐ Yes (1)
Q43	Do you think this kit should be sold conveniently in public outlets?	☐ No (0) ☐ Yes (1)
Q44	Would you recommend this kit to other people?	☐ No (0) ☐ Yes (1)
Q45	What is the maximum amount you are willing to pay for a rapid test kit (in Singapore dollars) (Usual HIV test costs about $20)?	☐ Less than $10 (1) ☐ $11 - $20 (2) ☐ $21 - $30 (3) ☐ $31 - $40 (4) ☐ $41 - $50 (5) ☐ More than $50 (6)
Q46	(Please write your comments and suggestions on the rapid test kit):	

Thank you for your participation

Tan Tock Seng HOSPITAL

HIV Rapid Test Questionnaire (POST-TEST)

Subject Study ID ☐

FOR STAFF COMPLETION (Please tick ☑ only one choice unless specified)

Q47	**The patient is a diagnosed HIV positive case?**	☐ No (0) ☐ Yes (1)
Q48	**The participant performed all the steps in the test correctly?**	☐ No (0) ☐ Yes (1)

If NO to Q48

Q48a **Which steps were wrong?**

- ☐ Opening of rapid test kit package (1)
- ☐ Removal of solution bottle from package (2)
- ☐ Removal of solution bottle cap (3)
- ☐ Placing of solution bottle on stand (4)
- ☐ Removal of swab stick from package (5)
- ☐ Holding of swab stick (6)
- ☐ Swabbing of upper gums (7)
- ☐ Swabbing of lower gums (8)
- ☐ Dipping of swab stick into solution bottle (9)
- ☐ Leaving swab stick to stand in solution bottle for at least 20 mins (10)
- ☐ Interpretation of results after 20 mins, but within 40 mins (11)

The participant read the following sample results correctly:

Q49	**Positive**	☐ Yes (1)<br>☐ Read as "Invalid" (3)	☐ Read as "Negative" (2)
Q50	**Negative**	☐ Yes (1)<br>☐ Read as "Invalid" (3)	☐ Read as "Positive" (2)
Q51	**Control absent (Invalid)**	☐ Yes (1)<br>☐ Read as "Positive" (3)	☐ Read as "Negative" (2)
Q52	**Lines not within range (Invalid)**	☐ Yes (1)<br>☐ Read as "Positive" (3)	☐ Read as "Negative" (2)

Outcome of the rapid test results performed by:

Q53	**Participant**	☐ Positive (1)<br>☐ Invalid (3)	☐ Negative (2)
Q54	**Research Assistant**	☐ Positive (1)<br>☐ Invalid (3)	☐ Negative (2)
Q56	**Date of confirmatory test result**	☐☐ / ☐☐ / ☐☐ (dd/mm/yy)	
Q56a	**Outcome of the confirmatory test result**	☐ Positive (1)<br>☐ Intermediate (3)	☐ Negative (2)
Q57	**Remarks (if any)**	☐	
Q58	**Name of Staff administering questionnaire**	☐	
Q59	**Signature of Staff administering questionnaire**	☐	
Q60	**Date of questionnaire administered**	☐☐ / ☐☐ / ☐☐ (dd/mm/yy)	
