# Supplementary material for: Accuracy and User-Acceptability of HIV Self-Testing Using an Oral Fluid-Based HIV Rapid Test
Source: PLoS One. 2012 Sep 17;7(9):e45168. doi: 10.1371/journal.pone.0045168 (PMC3444491; doi:10.1371/journal.pone.0045168)
Supplement: Appendix S2 — Patient Instruction Sheet. (PDF) [file pone.0045168.s002.pdf]

RAPID HIV 1/2 ANTIBODY TEST (ORAL)

ITEM LIST FOR RAPID TEST KIT

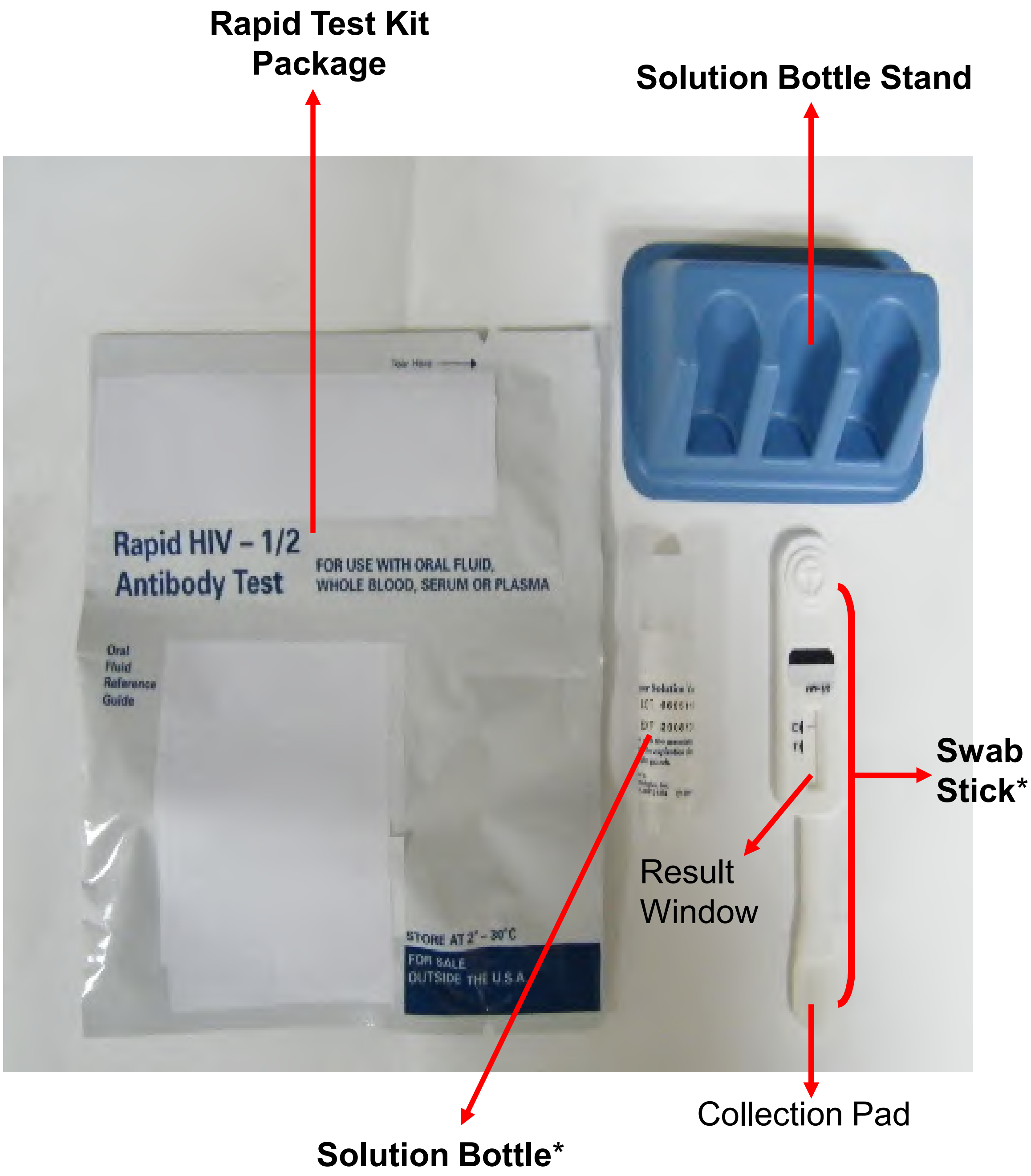

**Swab-stick and solution bottle are found in the Rapid Test Kit Package*

RAPID HIV 1/2 ANTIBODY TEST (ORAL)

USER INSTRUCTION SHEET

Please read instructions carefully before performing test

1. Open pack by tearing the ridge from top to bottom.

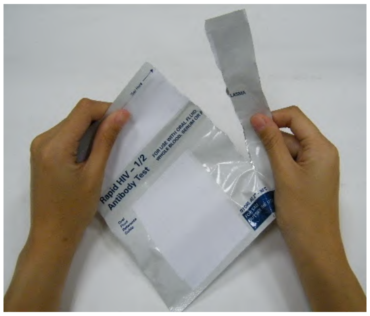

2. Remove solution bottle from package.

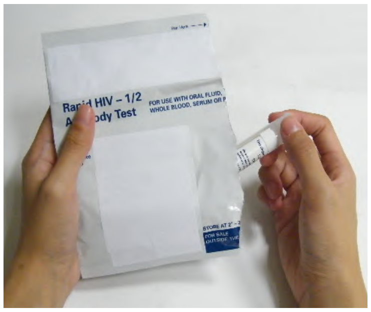

3. Remove the bottle cap carefully, and avoid spilling.

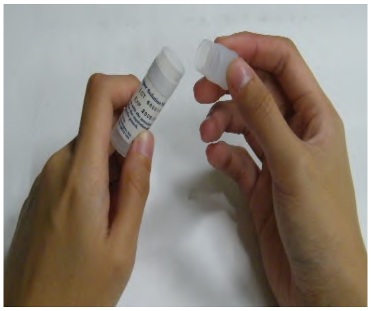

4. Place solution bottle on the solution bottle stand.

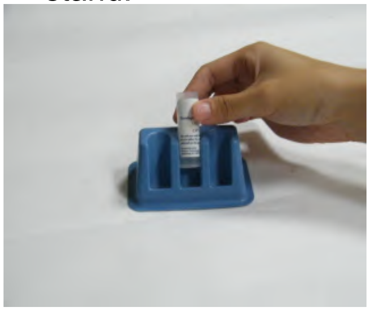

5. Remove swab stick from package.

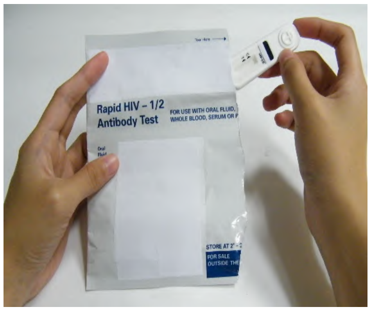

6. Hold swab stick as shown below. Do not touch the collection pad.

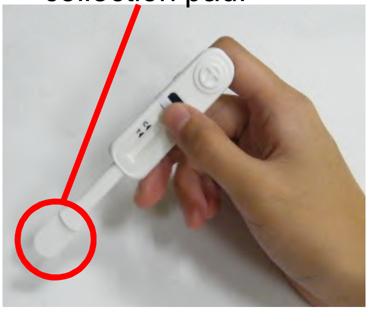

7. Swab around the upper gums with the collection pad, but avoid tongue, cheeks and other parts of your mouth.

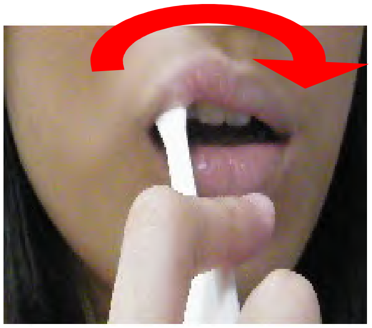

8. Swab around the lower gums with the collection pad, but avoid tongue, cheeks and other parts of your mouth.

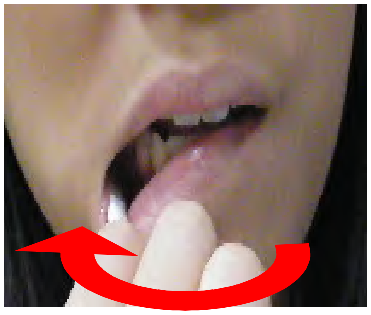

9. Place swab stick into the solution bottle.

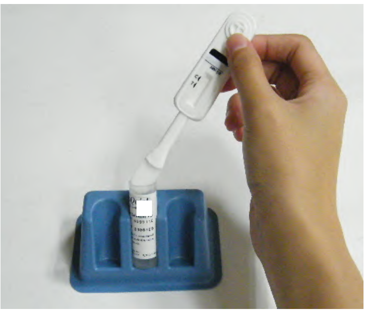

RAPID HIV 1/2 ANTIBODY TEST
(ORAL)

USER INSTRUCTION SHEET

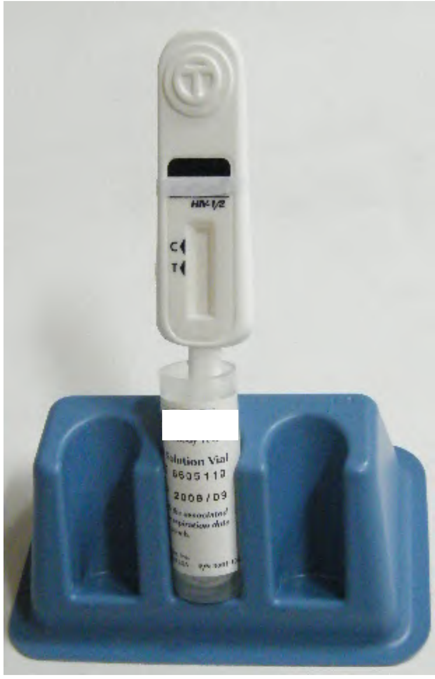

10. Let the swab stick stand in the solution bottle for 20mins.

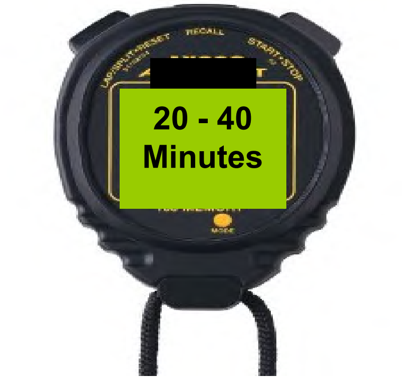

11. Read results after 20 minutes, but not more than 40 minutes.

Please turn to the next page for information on how to interpret your results.

RAPID HIV 1/2 ANTIBODY TEST (ORAL)

RESULTS INTERPRETATION SHEET

INVALID

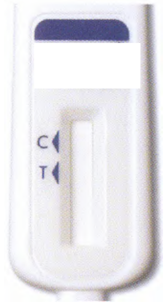

No line seen

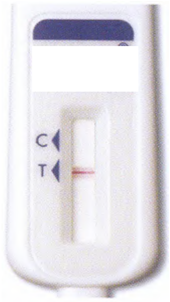

Line next to triangle labeled **T**, no line at **C**.

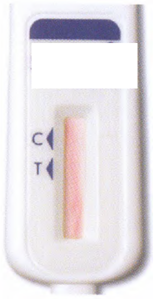

Pink-Reddish coloration seen throughout the result window

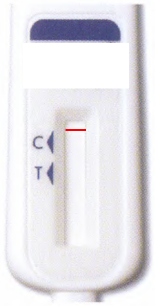

Line not inside triangle, either above **C** or **T**

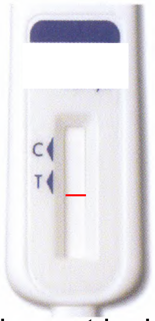

Line not inside triangle, either below **C** or **T**

NEGATIVE

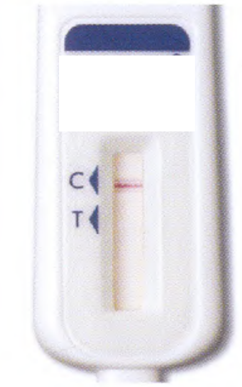

Line next to the triangle labeled **C.**

POSITIVE

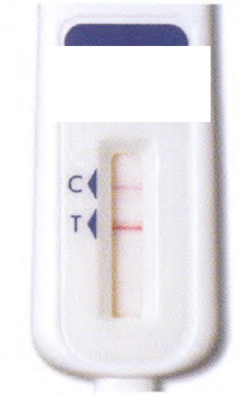

Two lines seen (one next to the triangle labeled **C** and one next to the triangle labeled **T**), no matter how faint these lines are**.**
